# Supplementary material for: Regulatory T Cells Boost Efficacy of Post-Infarction Pluripotent Stem Cell-Derived Cardiovascular Progenitor Cell Transplants
Source: Cells. 2025 Jun 23;14(13):956. doi: 10.3390/cells14130956 (PMC12248464; doi:10.3390/cells14130956)
Supplement: Supplementary file 1 [file cells-14-00956-s001.zip › cells-3656457-supplementary.pdf]

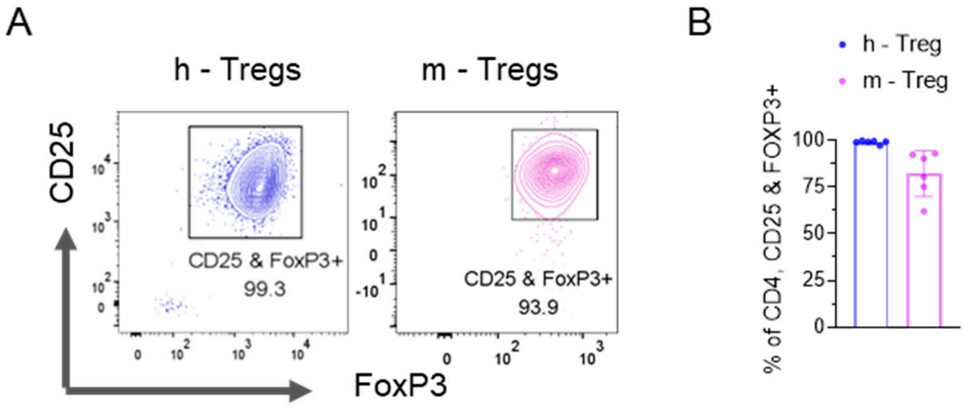

Supplementary Figure S1. Human and murine T regulatory cells display T regulatory cells markers after In vitro culture. A) Representative flow cytometry plots for the phenotyping of human and murine T regulatory cells are shown in the left and right contour plots, respectively, at the end of the in vitro culture period. CD25 expression is represented on the y-axis and FoxP3 on the x-axis. **B)** The graph displays the mean of the percentage of cells positive for CD4, CD25, and FoxP3 out of the live cells gate at the end of the In vitro culture period for human and murine T regulatory cells, represented by blue and purple bars, respectively, across all six independent cell batches used throughout the In vitro and In vivo experiments. Abbreviations: h - Tregs (human T regulatory cells), m – Tregs (murine T regulatory cells).

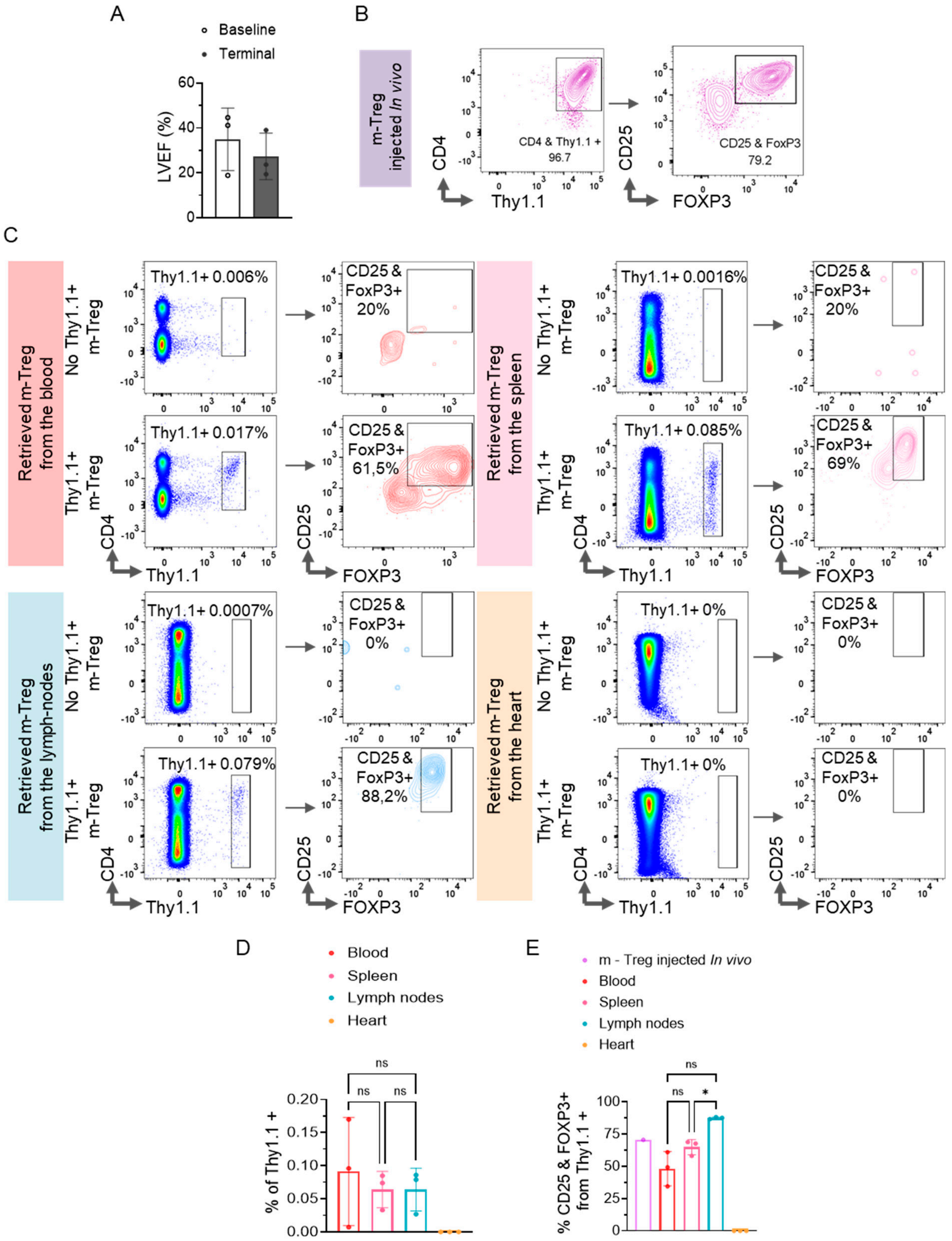

Supplementary Figure S2. Thy1.1 + Tregs are detectable in the blood, spleen, and lymph nodes two weeks following tail vein injection in infarcted mice. A) Left ventricle ejection

fraction measured via echocardiography at 24 hours (baseline) and five weeks (terminal) after the myocardial infarction. **B)** The representative plot illustrates the flow cytometric analysis of CD4 (y-axis), Thy1.1 (y-axis), CD25 (y-axis), and FoxP3 (x-axis) in *in vitro* cultured murine T regulatory cells prior to their intravenous injection into infarcted mice. **C)** Flow cytometric analysis was conducted to assess CD4 (y-axis), Thy1.1 (y-axis), CD25 (y-axis), and FoxP3 (x-axis) in cells retrieved from the blood, spleen, lymph nodes, and heart of animals sacrificed two weeks after receiving injections of Thy1.1+ murine T regulatory cells. **D)** Quantification of the percentage of Thy1.1+ cells detected by flow cytometry in the cells isolated from the blood, spleen, lymph nodes and heart. **E)** Two weeks post-adoptive transfer, we quantified CD25 and FoxP3+ cell percentages within the Thy1.1+ population from injected cells and those retrieved from blood, spleen, lymph nodes, and heart of euthanized mice. Three infarcted mice were injected with Thy1.1+ murine T regulatory cells. *P<0.05; **P<0.01; ***P<0.001. One-way ANOVA multiple comparisons analysis assuming equal variance was applied. Abbreviations: h - Tregs (human T regulatory cells), m – Tregs (murine T regulatory cells), LVEF (left ventricle ejection fraction).
